# Serum testosterone levels are positively associated with serum anti-mullerian hormone levels in infertile women

**DOI:** 10.1038/s41598-021-85915-x

**Published:** 2021-03-18

**Authors:** Li-Te Lin, Chia-Jung Li, Kuan-Hao Tsui

**Affiliations:** 1 Department of Obstetrics and Gynecology, Kaohsiung Veterans General Hospital, No.386, Dazhong 1st Rd., Zuoying Dist., Kaohsiung City, 81362 Taiwan; 2 Institute of BioPharmaceutical Sciencesciences, National Sun Yat-Sen University, Kaohsiung City, Taiwan; 3 Department of Obstetrics and Gynecology, National Yang-Ming University School of Medicine, Taipei, Taiwan; 4 Department of Pharmacy and Master Program, College of Pharmacy and Health Care, Tajen University, Pingtung County, Taiwan

**Keywords:** Infertility, Predictive markers

## Abstract

Anti-Mullerian hormone (AMH) and testosterone (T) both play distinct roles in the early stages of folliculogenesis. However, the relationship between serum T and AMH levels is poorly understood. This study aimed to investigate the association between serum T and AMH levels in infertile women. A total of 1935 infertile women aged 20–46 years were included in the cross-sectional study and divided into four quartile groups (Q1 to Q4) based on serum T levels. Compared to the subjects in the highest T quartile (Q4), those in the lowest T quartile (Q1) showed significantly lower AMH levels. After adjustment for age, body weight, body mass index and FSH, increasing T quartile categories were associated with higher AMH levels. Binary logistic regression analyses revealed that the odds for the risk of diminished ovarian reserve (DOR) were 11.44-fold higher in Q1 than in Q4 and the odds for the risk of excess ovarian reserve (EOR) were 10.41-fold higher in Q4 than in Q1. Our data show that serum T levels are positively associated with serum AMH levels and suggest that androgen insufficiency may be a potential risk factor for DOR; androgen excess may lead to EOR in infertile women.

## Introduction

Anti-Mullerian hormone (AMH), a glycoprotein belonging to the transforming growth factor β superfamily, is generated by granulosa cells (GCs) of growing follicles from the primary to the small antral follicles in the ovary^1^. The expression of AMH increases prior to follicle-stimulating hormone (FSH)-dependent selection (follicles up to 8 mm) and rapidly decreases after FSH-dependent selection (follicles > 8 mm)^2^. Serum AMH levels reflect the pool of growing follicles and thus are currently well known as a reliable biomarker for functional ovarian reserve^3^. Serum AMH levels gradually decline with age from the age of 25 years onward^4,5^. Serum AMH levels aid in the prediction of ovarian responses to controlled ovarian hyperstimulation^6,7^ and may be used in the individualization of starting doses of gonadotropin^8,9^. However, little is known about possible factors that affect serum AMH concentrations.

Androgens have been described to be involved in follicle recruitment and promotion of follicle growth^10,11^. The major circulating androgens in women include dehydroepiandrosterone sulfate (DHEA-S), dehydroepiandrosterone (DHEA), androstenedione, testosterone (T) and dihydrotestosterone (DHT). T and DHT, which are generated equally from the ovary and adrenals, are the only bioactive androgens that directly bind to the androgen receptor (AR)^12^. Similar to AMH, the action of T primarily occurs during the early stages of folliculogenesis because AR is the most abundant in the GCs of small follicles^13^. Peak serum T levels are achieved in early adulthood and show a decline with age as AMH^14^. Furthermore, studies have revealed that T supplementation may have a positive effect on antral follicle count (AFC) in poor ovarian responders (PORs)^15,16^ and T suppression by oral contraceptives might decrease the number of antral follicles and even serum AMH levels in polycystic ovary syndrome (PCOS) patients^17,18^.

Based on the abovementioned findings, we hypothesized that serum levels of T had a positive association with serum AMH levels. However, few large-scale studies have examined the relationship between serum T and AMH levels. Therefore, we conducted a large cross-sectional study in infertile women to clarify the association between serum T and AMH concentrations.

## Results

The characteristics of the 1935 infertile women included in the study are shown in Table 1. The average age was 35.1 ± 4.7 years (range 21–46 years); the average body mass index (BMI) was 22.4 ± 3.8 kg/m^2^ (range 14.7–40.8 kg/m^2^). The mean serum T level was 0.33 ± 0.35 ng/mL (range 0.05–4.81 ng/mL) and the mean serum AMH level was 3.6 ± 2.8 ng/mL (range 0.03–22.08 ng/mL). Infertility causes included tubal factor (12.1%), male factor (11.6%), diminished ovarian reserve (13.0%), PCOS (9.6%), endometriosis (13.8%), uterine factor (8.0%), multiple factors (21.7%) and unexplained infertility (10.1%). In the group of women < 35 years (n = 887), the mean serum T level was 0.35 ± 0.31 ng/mL and the mean serum AMH level was 4.7 ± 3.1 ng/mL. In women ≥ 35 years (n = 1048), the average serum T level was 0.31 ± 0.38 ng/mL, and the average serum AMH level was 2.6 ± 2.0 ng/mL.

**Table 1:** The characteristics of the study population.

Variables	All population (n = 1935)	Age < 35 years (n = 887)	Age ≥ 35 years (n = 1048)
Age (years)	35.1 ± 4.7	30.9 ± 2.7	38.6 ± 2.8
Body height (cm)	160.5 ± 5.8	160.8 ± 5.8	160.3 ± 5.7
Body weight (kg)	57.7 ± 10.5	56.8 ± 10.6	58.5 ± 10.3
BMI (kg/m^2^)	22.4 ± 3.8	22.0 ± 3.9	22.7 ± 3.8
TSH (μIU/mL)	1.7 ± 1.4	1.7 ± 1.8	1.7 ± 1.1
Prolactin (ng/mL)	15.6 ± 15.3	15.4 ± 12.0	15.7 ± 17.7
25-OH-vitamin D (ng/mL)	21.8 ± 6.8	21.1 ± 6.2	22.2 ± 7.2
FSH (mIU/mL)	5.2 ± 3.3	4.8 ± 2.5	5.6 ± 3.8
Testosterone (ng/mL)	0.33 ± 0.35	0.35 ± 0.31	0.31 ± 0.38
DHEA-S (μg/dL)	239.5 ± 114.2	262.0 ± 107.7	220.1 ± 116.1
AMH (ng/mL)	3.6 ± 2.8	4.7 ± 3.1	2.6 ± 2.0
**Causes of infertility**			
Tubal factor	12.1%(235/1,935)	15.1%(134/887)	9.6%(101/1,048)
Male factor	11.6%(225/1,935)	11.2%(99/887)	12.0%(126/1,048)
DOR*	13.0%(252/1,935)	4.5%(40/887)	20.2%(212/1,048)
PCOS*	9.6%(186/1,935)	12.1%(107/887)	7.5%(79/1,048)
Endometriosis	13.8%(267/1,935)	15.3%(136/887)	12.5%(131/1,048)
Uterine factor	8.0%(155/1,935)	9.9%(88/887)	6.4%(67/1,048)
Multiple factors	21.7%(420/1,935)	16.5%(146/887)	26.1%(274/1,048)
Unexplained infertility	10.1%(195/1,935)	15.4%(137/887)	5.5%(58/1,048)

Data are presented as the mean ± standard deviation.

*BMI* body mass index, *TSH* thyroid-stimulating hormone, *FSH* follicle stimulation hormone, *DHEA-S* dehydroepiandrosterone sulfate, *AMH* anti-Müllerian hormone.

*DOR, diminished ovarian reserve, was defined as AMH < 1.2 ng/mL based on POSEIDON criteria.

*PCOS, polycystic ovarian syndrome, was determined by Rotterdam criteria.

The subjects were then categorized into four quartile groups (Q1 to Q4) based on serum T concentrations (Table 2). AMH, DHEA-S, body weight and BMI were positively associated with the T quartile category, whereas age linearly decreased as the T quartile category rose from Q1 to Q4 (all *p* for tend < 0.001). Body height, TSH, prolactin, 25-OH-vitamin D and FSH were not significantly different among the T quartile categories. Furthermore, compared to the AMH levels in the subjects in the highest T quartile (Q4), those in the lower T quartile (Q1, Q2 and Q3) demonstrated significantly lower AMH levels (*p* < 0.05).

**Table 2:** Clinical characteristics according to serum testosterone quartile categories.

Variables	Quartile of serum testosterone				
	Q1 (n = 481)	Q2 (n = 491)	Q3 (n = 478)	Q4 (n = 435)	*p* for tend
Testosterone (ng/mL)	≤ 0.21	0.22 ~ 0.27	0.28 ~ 0.35	≥ 0.36	
Age (years)	36.9 ± 4.5*	35.4 ± 4.6*	34.6 ± 4.5*	33.3 ± 4.5	< 0.001
Body height (cm)	160.5 ± 5.7	160.4 ± 5.6	160.4 ± 5.7	160.7 ± 6.0	0.723
Body weight (kg)	56.9 ± 9.2*	56.4 ± 9.5*	57.2 ± 10.2*	60.9 ± 12.5	< 0.001
BMI (kg/m^2^)	22.1 ± 3.3*	21.9 ± 3.4*	22.2 ± 3.9*	23.6 ± 4.6	< 0.001
TSH (μIU/mL)	1.7 ± 1.0	1.7 ± 1.9	1.6 ± 1.0	1.7 ± 1.6	0.743
Prolactin (ng/mL)	14.4 ± 11.1	16.5 ± 21.8	15.3 ± 10.2	16.0 ± 15.2	0.248
Vitamin D (ng/mL)	21.5 ± 7.5	21.5 ± 6.8	21.7 ± 6.2	22.4 ± 6.5	0.141
FSH (mIU/mL)	5.4 ± 3.6	5.1 ± 3.1	5.1 ± 3.1	5.2 ± 3.3	0.344
DHEA-S (μg/dL)	170.3 ± 70.1*	221.4 ± 76.0*	260.3 ± 96.9*	319.1 ± 151.0	< 0.001
AMH (ng/mL)	2.1 ± 1.5*	3.3 ± 2.1*	3.9 ± 2.7*	5.6 ± 3.7	< 0.001

Data are presented as the mean ± standard deviation.

*p* values for trends were generated by linear regression analysis.

*Statistically significantly different from the highest quartile category (Q4) using Bonferroni’s method in an analysis of variance (ANOVA) test.

*BMI* body mass index, *TSH* thyroid-stimulating hormone, *Vitamin D* 25-OH-vitamin D, *FSH* follicle stimulation hormone, *DHEA-S* dehydroepiandrosterone sulfate, *AMH* anti-Müllerian hormone.

**Figure 1:**
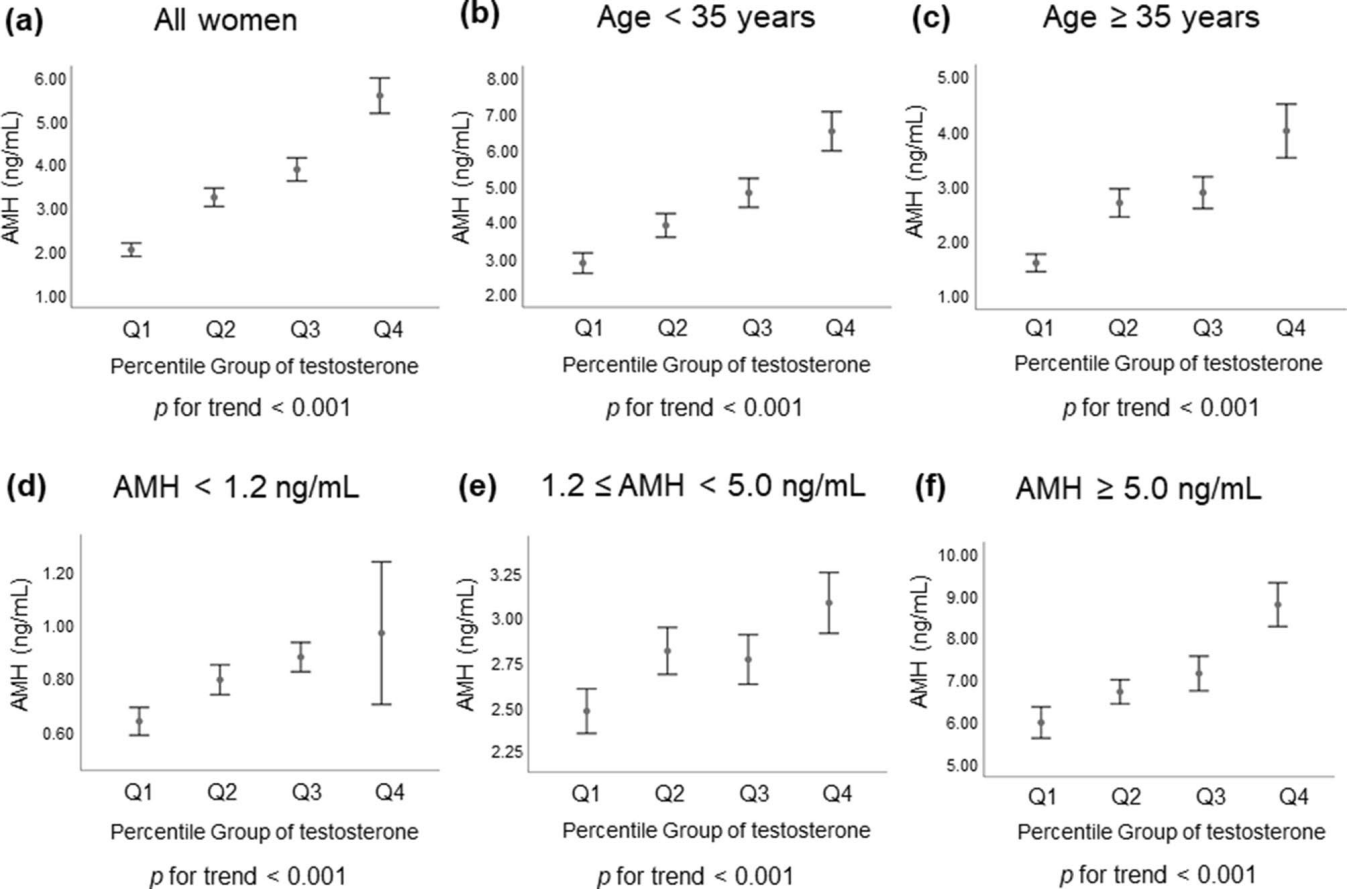
Serum AMH levels according to serum testosterone quartile categories in different groups. (**a**) all women, (**b**) age < 35 years, (**c**) age ≥ 35 years, (**d**) AMH < 1.2 ng/mL, (**e**) 1.2 ≤ AMH < 5.0 ng/mL and (**f**) AMH ≥ 5.0 ng/mL. The *p* values for the trends were generated using generalized linear models after adjustment for multivariate confounders. Multivariable confounders included age, weight, body mass index, and FSH. Error bar represented 95% confidence interval of AMH. *AMH* anti-Mullerian hormone, *Q* quartile.

Generalized linear models were used to assess the independent association of serum T quartile categories with AMH levels after adjusting for potential confounding factors, including age, body weight, BMI and FSH. Regardless of all women or different age groups (< 35 or ≥ 35 years) or different AMH groups (< 1.2, 1.2–5.0, or ≥ 5.0 ng/mL), AMH levels significantly increased in a dose-dependent fashion across increasing T quartile categories in the multivariate adjustment model as shown in Fig. 1.

**Figure 2:**
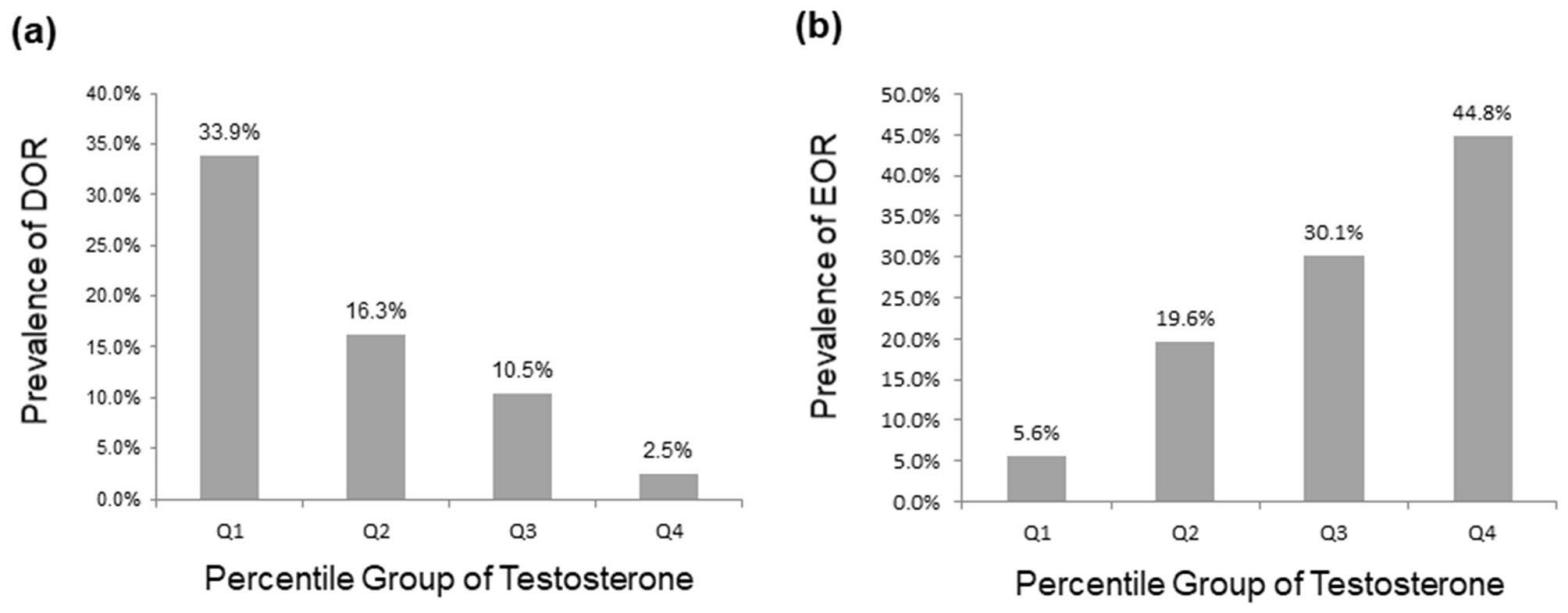
Prevalence of (**a**) diminished ovarian reserve (DOR) and (**b**) excess ovarian reserve (EOR) according to serum T quartile categories. DOR was defined as serum AMH levels < 1.2 ng/mL; serum AMH levels ≥ 5.0 ng/mL were defined as EOR. *Q* quartile.

The overall proportions of subjects who met the criteria for diminished ovarian reserve (DOR, AMH < 1.2 ng/mL) and excess ovarian reserve (EOR, AMH ≥ 5.0 ng/mL) were 16.3% (242/1485) and 24.2% (359/1485), respectively. The prevalence of DOR among subjects in Q1, Q2, Q3 and Q4 was 33.9% (128/378), 16.3% (65/398), 10.5% (41/392) and 2.5% (8/317), respectively (Fig. 2a). Multiple logistic regression analyses revealed that the ORs for the risk of DOR dose-dependently increased across decreasing T quartile categories, and the odds for the risk of DOR were 11.44-fold higher in subjects in Q1 than in those in Q4 after adjustment for potential confounders (Table 3). The prevalence of EOR among subjects in Q1, Q2, Q3 and Q4 was 5.6% (21/378), 19.6% (78/398), 30.1% (118/392) and 44.8% (142/317), respectively (Fig. 2b). Multiple logistic regression analyses revealed that the ORs for the risk of EOR dose-dependently increased across increasing T quartile categories, and the odds for the risk of EOR were 10.41-fold higher in subjects in Q4 than in those in Q1 after adjustment for potential confounders (Table 3).

**Table 3:** Risk for diminished ovarian reserve (DOR) or excess ovarian reserve (EOR) according to serum testosterone quartile categories.

Variables	Risk for DOR (AMH < 1.2 ng/mL)		Risk for EOR (AMH ≥ 5.0 ng/mL)	
	Adjusted OR (95% CI)	*p* value	Adjusted OR (95% CI)	*p* value
**T quartile categories**				
Q1 (0.05 ~ 0.21 ng/mL)	11.44 (4.75–27.53)	< 0.001	1.00 (Ref.)	
Q2 (0.22 ~ 0.27 ng/mL)	5.45 (2.23–13.33)	< 0.001	3.74 (2.09–6.71)	< 0.001
Q3 (0.28 ~ 0.35 ng/mL)	3.12 (1.23–7.88)	0.016	6.54 (3.70–11.54)	< 0.001
Q4 (0.36 ~ 4.81 ng/mL)	1.00 (Ref.)		10.41 (5.84–18.55)	< 0.001

ORs (95% CI) for DOR or EOR according to serum testosterone quartile categories after adjustment for multivariate confounders in infertile women (n = 1,935). Binary logistic regression analysis was performed. Multivariate confounders included age, weight, body mass index, and FSH.

*T* testosterone, *AMH* anti-Müllerian hormone, *OR* odds ratio, *CI* confidence interval, *Q* quartile, *Ref.* reference.

The age-dependent distribution of serum T levels in all patients (n = 1885) is shown in Table 4. The median serum T level in all patients was 0.27 ng/mL; the median serum T levels in the 20–25, 26–30, 31–35, 36–40 and 41–46 age groups were 0.36, 0.32, 0.28, 0.26 and 0.23 ng/mL, respectively.

**Table 4:** Serum testosterone level distribution (ng/mL) based on age.

Age (years)	Mean	Percentile					n
		5^th^	10^th^	Median	90^th^	95^th^	
20 ~ 25	0.38	0.20	0.21	0.36	0.63	0.76	35
26 ~ 30	0.40	0.17	0.21	0.32	0.56	0.89	288
31 ~ 35	0.34	0.16	0.18	0.28	0.47	0.54	688
36 ~ 40	0.32	0.15	0.17	0.26	0.42	0.51	627
41 ~ 46	0.27	0.13	0.15	0.23	0.37	0.45	247
All	0.33	0.15	0.17	0.27	0.46	0.55	1,885

## Discussion

To the best of our knowledge, the present study is the largest clinical study to investigate the association between serum T and AMH levels in infertile women. In this large retrospective cross-sectional study of 1935 infertile women, higher serum T concentrations were associated with higher serum AMH levels after adjustment for potential confounders. Consistently, infertile women in the lowest T quartile had a 11.44-fold higher odds for the risk of DOR than those in the highest T quartile. The odds for the risk of EOR were 10.41-fold higher in infertile women in the highest T quartile than in those in in the lowest T quartile.

Androgens play important roles in the regulation of ovarian function. AR, expressed in oocytes, GCs and theca cells, is pivotal for normal follicular development^10,11^. AR is most highly expressed in the GCs of preantral and early antral follicles, and its expression decreases as the follicles grow^13^. Via the AR, androgens increase the FSH receptor and synergize with FSH to enhance follicle growth^19–21^. Moreover, androgens support follicle health by decreasing follicle atresia and GC apoptosis, and stimulating the proliferation and differentiation of GC^20,22,23^. Although AR is not expressed in primordial follicles, androgens promote primordial follicle initiation^24,25^ via indirect mechanisms, such as upregulation of insulin-like growth factor 1 expression^25^. The above information supports our results that lower T levels were associated with a higher risk of DOR. Studies have shown that women with DOR or POI demonstrated significantly lower serum T levels than controls^26,27^. On the other hand, androgen excess may lead to impaired ovarian function and dysregulated follicle development, displaying irregular cycles, oligo-ovulation and polycystic ovaries^28,29^. These findings agree with our results that infertile women with higher T levels had a higher risk of EOR. Thus, an optimal balance in androgenic actions is necessary for maintaining normal ovarian function. Serum T concentrations decline with age^14^. Thus, our study demonstrated an age-specific normal reference range for serum T levels to aid in identifying women who suffer from androgen insufficiency or excess (Table 4).

As mentioned above, androgens enhance FSH activity through increased FSH receptor expression^20,21^. FSH stimulates AMH expression^30,31^, which could inhibit the sensitivity of preantral follicles to FSH to avoid premature selection by FSH in the gonadotrophin-independent stage^32,33^. Therefore, Dewailly et al. proposed that androgens may promote AMH generation via enhancement of FSH-stimulated AMH expression^34^. Elevated AMH could attenuate FSH-induced aromatase activity, leading to an increase in androgen levels^33^. Moreover, via AMH receptor type 2 on the hypothalamus and pituitary, elevated AMH may boost GnRH-dependent LH pulsatility and secretion which stimulates androgen production in theca cells^35,36^. Taken together, it seems that androgens and AMH mutually stimulate each other. These results support our results that serum T concentrations positively correlated with serum AMH levels. Some studies also showed a positive correlation between serum androgens and AMH^37–39^. However, some studies revealed contradictory results, which indicated that androgens or FSH may have an inhibitory effect on AMH expression^40–42^. Thus, the accurate relationship between androgens and AMH remains unclear. Further studies with ideal experimental models are needed to clarify the relationship.

Serum T levels have been suggested to be positively associated with ovarian response^43,44^ and even pregnancy outcomes^44,45^ in women undergoing IVF cycles. Although some conflicting studies have shown that serum T levels do not predict IVF outcomes^43,46^, available data have indicated that T supplementation may improve ovarian response and IVF outcomes in PORs^47,48^. In a randomized controlled trial of 110 PORs undergoing IVF cycles, Kim et al. reported that pretreatment with transdermal T gel significantly increased AFC and reduced the day of stimulation and total dosage of gonadotropins. In addition, the numbers of oocytes retrieved, mature oocytes, fertilized oocytes, and good-quality embryos were significantly higher in the T pretreatment group than in the control group^16^. A meta-analysis of 7 randomized controlled trials conducted by Noventa and colleagues revealed that PORs receiving T therapy demonstrated higher numbers of total oocytes, MII oocytes and total embryos, as well as a higher clinical pregnancy rate and live birth rate than controls^47^. On the other hand, the addition of insulin sensitizing agents to suppress insulin resistance and excess androgen may ameliorate the results of ovulation induction in PCOS patients^49,50^. In the present study, we provided an age-specific normal reference range for serum T levels to help determine whether infertile women require agents for androgen enhancement or suppression (Table 4). Further large-scale, well-defined randomized controlled trials are still mandatory to confirm the effectiveness of androgen supplementation and androgen suppression by agents.

Several potential limitations should be taken into consideration when interpreting the data. First, the retrospective design of this study presented the major limitation. Second, since this is a cross-sectional study, a causal relationship could not be determined between serum T and AMH levels. Third, our study population only consisted of infertile women. We cannot be sure that our results would be applicable to the general population. Fourth, direct testosterone immunoassays have limitations for clinical use, particularly for low concentrations found in women and children^51^. This would be a relevant source of bias. Although high correlation (*r* ≥ 0.95) of serum T levels was observed between the ARCHITECT 2nd Generation Testosterone assay and the LCMS, the bias could not be totally excluded. Fifth, serum concentrations of free T, androstenedione, and sex hormone-binding globulin (SHBG) were not measured in our routine infertility evaluation. Thus, accurate androgen status would be uncertain in this study.

In conclusion, our data reveal an obvious positive association between serum T and AMH levels in infertile women. Additionally, the risk of DOR was significantly increased in a dose-dependent manner across decreasing T quartile categories; the risk of EOR dose-dependently increased across increasing T quartile categories. Long-term longitudinal studies are required to confirm our results.

## Methods

### Study design and participants

This was a retrospective cross-sectional study. Infertile women were first identified based on the International Classification of Diseases, Ninth Revision, Clinical Modification (ICD-9-CM), code 628, from the clinical database in Kaohsiung Veterans General Hospital. To avoid any potential misclassifications, among the infertile women identified by the ICD-9-CM code, only subjects who received a complete infertility survey in the reproductive center of Kaohsiung Veterans General Hospital were selected. A total of 2476 infertile women were identified from May 2013 through March 2020. Then, we performed the chart review of these 2476 infertile women and selected the women who truly met the definition of infertility among them. Infertility was defined by the failure to achieve a successful pregnancy after 12 months or more of regular, unprotected sexual intercourse^52^. Moreover, we excluded the following subjects based on chart review: (1) subjected who experienced repeated surveys; (2) subjects who had extreme age (< 20 or > 46 years) (3) subjected who was diagnosed as primary ovarian insufficiency; (4) subjects who ever underwent ovarian surgery; (5) subjected who had a history of exposure to cytotoxic agents or pelvic irradiation for malignancy; (6) subjected who had androgen-secreting tumors; (7) subjected who was diagnosed as congenital adrenal hyperplasia (8) subjects who had androgen supplementation or hormonal therapy during the previous 3 months. A total of 1935 infertile women were finally included in the study. The study protocol was approved by the institutional review board in Kaohsiung Veterans General Hospital, with the identifier KSVGH20-CT11-03, and conformed to the “Declaration of Helsinki for Medical Research involving Human Subjects.” Need of informed consent was waived by the institutional review board in Kaohsiung Veterans General Hospital due to the retrospective design.

### Biochemical measurements

For the infertility survey, we checked blood examinations including AMH, T, DHEA-S, FSH, luteinizing hormone (LH), estradiol, thyroid-stimulating hormone (TSH), prolactin and 25-OH-vitamin D levels. Serum AMH levels were measured by chemiluminescent immunoassay using the Access Immunoassay Systems, the Beckman Coulter enzyme-linked immunoassay (Beckman Coulter, Marseille, France). The analytical range of the lower limit of detection was 0.02 ng/mL. The intra-assay coefficient of variation (CV) was 3.0%, and the interassay CV was 7.0%. DOR was defined as serum AMH levels < 1.2 ng/mL based on the POSEIDON criteria^53^; serum AMH levels ≥ 5.0 ng/mL, modified from the revised Rotterdam criteria^54^, were considered EOR in this study.

Serum T was measured by chemiluminescent microparticle immunoassay using the ARCHITECT 2nd Generation Testosterone assay (Abbott GmbH, Max-Planck-Ring 2, Wiesbaden, Germany). The range was 0.04 ng/mL to 18.62 ng/mL. The assay had a limit of quantitation of ≤ 0.04 ng/mL and had a within-laboratory imprecision of ≤ 10% CV. Potential interference in the ARCHITECT 2nd Generation Testosterone assay from hemoglobin, bilirubin, triglycerides, protein and biotin was evaluated to be ≤ 10%. This assay had a correlation coefficient (*r*) of ≥ 0.95 for samples with testosterone concentrations ranging from 0.04 ng/mL to 10.09 ng/mL when compared to Liquid Chromatography-Tandem Mass Spectrometry (LCMS).

Serum DHEA-S, FSH, LH, estradiol, TSH, prolactin and 25-OH-vitamin D concentrations were measured by a chemiluminescent microparticle immunoassay on the ARCHITECT iSystem (Abbott, Longford, Ireland or Abbott, Wiesbaden, Germany). The total CV of these analyses were consistently < 3–10%. The specificity of these assays was determined by studying the cross-reactivity of structurally similar compounds. The cross-reactivity was calculated as a percent cross-reactivity. DHEA-S assay was shown to be 0% for testosterone, 0.003% for androstenedione, 0.025% for 19-hydroxyandrostenedione, 0.006% for DHEA glucuronide and 0.001% for estradiol. FSH assay was shown to be 0.002% for LH, 0.043% for TSH and 0.001% for hCG. LH assay was shown to be 0.01% for FSH, 0% for TSH and 0.01% for hCG. Estradiol assay was shown to be 0.1% for 17β-estradiol 3-sulfate and 0.7% for estrone. Prolactin assay was shown to be 0% for FSH, hCG, TSH and 0.001% for LH. TSH assay had an analytical specificity of < 10% cross reactivity with FSH, LH and hCG. 25-OH Vitamin D assay was shown to be 0.8% for vitamin D3 (Cholecalciferol), 0.4% for vitamin D2 (Ergocalciferol), 0.1% for 1,25-(OH)2-vitamin D3 and 0% for 1,25-(OH)2-vitamin D2. Blood samples were collected on the 2nd or 3rd day of the menstrual cycle. The reference intervals in the follicular phase were as follows: T, 0.14–0.53 ng/mL; DHEA-S, 35.0–430.0 µg/dL; FSH, 4–13 mIU/mL; LH, 1–18 mIU/mL; estradiol, 39–189 pg/mL; TSH, 0.35–4.94 μIU/mL; prolactin, 1.39–24.20 ng/mL; and 25-OH-vitamin D, 30 ~ 100 ng/mL.

### Statistical analysis

The normality of the distribution was tested using the Kolmogorov–Smirnov test. Continuous variables were presented as the mean ± standard deviation. The subjects were categorized into four quartile groups (Q1 to Q4) based on serum T concentrations. Quantitative variables were evaluated using the analysis of variance (ANOVA) and linear regression analysis among T quartile categories. Bonferroni’s method was used for post hoc pairwise comparison in the ANOVA test. Generalized linear model was performed to examine the correlation between serum AMH levels and T quartile categories after adjusting for potential confounders including age, weight, BMI and FSH. Odds ratios (ORs) and 95% confidence intervals (CIs) for DOR and EOR among T quartile categories were assessed using binary logistic regression after adjustment for potential confounders (age, weight, BMI and FSH). All analyses were conducted using statistical software, Statistical Package for Social Sciences (SPSS) version 20.0 (Chicago, IL, USA). All statistical tests used a two-tailed α of 0.05, and statistical significance was defined as *p* < 0.05.

### Ethics declarations

The study conformed to the ‘‘Declaration of Helsinki for Medical Research involving Human Subjects’’. Additionally, approval was obtained from the institutional review board at Kaohsiung Veterans General Hospital, with the identifier KSVGH20-CT11-03. The study was performed in accordance with approved guidelines. Need of informed consent was waived by the institutional review board in Kaohsiung Veterans General Hospital.

## Data Availability

The datasets generated during the current study are available from the corresponding author on reasonable request.
